# Supplementary material for: Complexome profiling of the Chlamydomonas psb28 mutant reveals TEF5 as an early PSII assembly factor
Source: Plant Cell. 2025 Mar 17;37(6):koaf055. doi: 10.1093/plcell/koaf055 (PMC12164590; doi:10.1093/plcell/koaf055)
Supplement: koaf055_Supplementary_Data [file koaf055_supplementary_data.zip › psb28_interactive.zip!/plots/intensity/qualityPlots/peptides/_Cre06.g296400.t1.2.html]

### Cre06.g296400.t1.2

Trivial name:   
SeqCoverage [median]: 24.70  
Mass: 45.38 kDa  
  
 

| Sequence | MissedCleavage | Mass [Da] | wtIntensity | muIntensity | Score |
| --- | --- | --- | --- | --- | --- |
| ANVLVVYAK | 0 | 975.58 | 1352.62 | 3593.09 | 147.52 |
| AYVYAVAAAADSGTPADR | 0 | 1767.84 | 0.00 | 581.65 | 32.93 |
| ELFKESSEGR | 1 | 1180.57 | 486.40 | 545.63 | 83.86 |
| GFSTAQKLDK | 1 | 1093.58 | 0.00 | 373.76 | 73.98 |
| GITAFLIEK | 0 | 990.57 | 0.00 | 3425.47 | 111.17 |
| GVAVMMSGLDYER | 0 | 1426.66 | 702.24 | 1318.91 | 100.88 |
| KQFGQAIGEFQLIQGK | 1 | 1790.97 | 0.00 | 2061.41 | 67.14 |
| LYEIGAGTSEIRR | 1 | 1463.77 | 0.00 | 1968.71 | 45.91 |
| TDPAAGPR | 0 | 783.39 | 893.36 | 2052.63 | 94.66 |
|  |  |  | 3434.6 | 15921.2 |  |
